# Does obesity affect acetabular cup position, spinopelvic function and sagittal spinal alignment? A prospective investigation with standing and sitting assessment of primary hip arthroplasty patients

**DOI:** 10.1186/s13018-021-02716-8

**Published:** 2021-10-26

**Authors:** Henryk Haffer, Zhen Wang, Zhouyang Hu, Luis Becker, Maximilian Müllner, Christian Hipfl, Matthias Pumberger, Yannick Palmowski

**Affiliations:** Center for Musculoskeletal Surgery, Charité - Universitätsmedizin Berlin, Corporate Member of Freie Universität Berlin, Humboldt-Universität Zu Berlin, and Berlin Institute of Health, Berlin, Germany

**Keywords:** Sagittal spinal alignment, Spinopelvic mobility, BMI, Dislocation, Hip replacement, Obesity

## Abstract

**Background:**

Total hip arthroplasty (THA) instability is influenced by acetabular component positioning, spinopelvic function and sagittal spinal alignment. Obesity is considered as a risk factor of THA instability, but the causal relationship remains unknown. This study aimed to investigate the influence of BMI on (1) spinopelvic function (lumbar flexibility, pelvic mobility and hip motion), (2) sagittal spinal alignment pre- and postoperatively and (3) acetabular cup position postoperatively in primary THA patients in a prospective setting.

**Methods:**

One hundred ninety patients receiving primary total hip arthroplasty were enrolled in a prospective cohort study and retrospectively analysed. All patients received stereoradiography (EOS) in standing and relaxed sitting position pre-and postoperatively. C7-sagittal vertical axis (C7-SVA), lumbar lordosis (LL), pelvic incidence (PI), pelvic tilt (PT), anterior plane pelvic tilt (APPT), and pelvic femoral angle (PFA) were assessed. Key parameters of the spinopelvic function were defined as lumbar flexibility (∆ LL = LL_standing_ − LL_sitting_), pelvic mobility (∆ PT = PT_standing_ − PT_sitting_) and hip motion (∆ PFA = PFA_standing_ − PFA_sitting_). Pelvic mobility was further defined based on ∆ PT as stiff, normal and hypermobile (∆ PT < 10°; 10°–30°; > 30°). The patients were stratified to BMI according to WHO definition: normal BMI ≥ 18.5–24.9 kg/m^2^ (n = 68), overweight ≥ 25.0–29.9 kg/m^2^ (n = 81) and obese ≥ 30–39.9 kg/m^2^ (n = 41). Post-hoc analysis according to Hochberg's GT2 was applied to determine differences between BMI groups.

**Results:**

Standing cup inclination was significant higher in the obese group compared to the normal BMI group (45.3° vs. 40.1°; *p* = 0.015) whereas standing cup anteversion was significantly decreased (22.0° vs. 25.3°; *p* = 0.011). There were no significant differences for spinopelvic function key parameter lumbar flexibility (∆ LL), pelvic mobility (∆ PT) and hip motion (∆ PFA) in relation to BMI stratified groups. The obese group demonstrated significant enhanced pelvic retroversion compared to the normal BMI group (APPT − 1.8° vs. 2.4°; *p* = 0.028). The preoperative proportion of stiff pelvic mobility was decreased in the obese group (12.2%) compared to normal (25.0%) and overweight (27.2%) groups. Spinal sagittal alignment in C7-SVA and PI-LL mismatch demonstrated significantly greater imbalance in the obese group compared to the normal BMI group (68.6 mm vs. 42.6 mm, *p* = 0.002 and 7.7° vs. 1.2°, *p* = 0.032, respectively) The proportion of patients with imbalanced C7-SVA was higher in the obese (58.5%) than in the normal BMI group (44.1%).

**Conclusions:**

The significantly increased spinal sagittal imbalance with altered pelvic mechanics is a potential cause for the reported increased risk of THA dislocations in obese patients. Consequently, the increased spinal sagittal imbalance in combination with normal pelvic mobility need to be taken into account when performing THA in obese patients.

**Supplementary Information:**

The online version contains supplementary material available at 10.1186/s13018-021-02716-8.

## Introduction

Total hip arthroplasty (THA) dislocations remain a leading cause of prosthesis failure and reoperation [1, 2]. Abnormal spinopelvic function has been reported as a contributing factor in the etiology of THA instability [3–8]. Consequently, spinopelvic function evaluated on standing and sitting radiographs has received attention by arthroplasty surgeons when attempting to preoperatively identify THA candidates with an increased risk for instability [9–11]. The spinopelvic function is represented by changes in posture from standing to sitting in the key spinopelvic complex parameters lumbar flexibility (∆ LL = LL_standing_–LL_sitting_), pelvic mobility (∆ PT = PT_standing_–PT_sitting_) and hip motion (∆ PFA = PFA_standing_–PFA_sitting_). Abnormal pelvic mobility is commonly classified as stiff with a change (∆ PT) of less than 10° and hypermobile with a change of more than 30° from standing to sitting respectively [12, 13]. It is reported that patients with restricted lumbar flexibility (∆ LL), stiff pelvic mobility (∆ PT) and increased hip motion (∆ PFA) have a significantly enhanced risk of THA dislocations and an inferior outcome [14–18]. However, not only stiff pelvic mobility, but also hypermobility is associated with poorer outcome and enhanced risk of THA dislocations [19]. Sagittal spinal malalignment is also linked to the spinopelvic complex, as the pelvis can compensate for spinal sagittal imbalance by pelvic retroversion to ensure an erect posture. This may alter acetabular orientation and involves the risk of posterior impingement with anterior THA dislocation [4, 20–24].

Some risk factors contributing to abnormal spinopelvic function have been identified, but for a widely common and increasing disease as obesity, the influence on the spinopelvic complex is poorly understood [25]. The rising relevance of obesity is emphasized by a study predicting that by 2030, 86.3% of the adults in the USA will be overweight or obese [26]. Since obesity is associated with osteoarthritis, obese patients are at increased risk of hip replacement and on average undergo this procedure 10 years earlier than normal weight patients [27–29]. However, overweight THA patients were shown to have an increased risk of infection, thromboembolic complications, aseptic loosening and prolonged hospitalization after arthroplasty [30]. There is some evidence that obesity increases the risk of THA instability, but the causal relationship between obesity and dislocation is still not known [31–33]. Previous investigations revealed inconsistent results in relation to spinopelvic alignment and the Body Mass Index (BMI) [34–36]. To the best of our knowledge, there is no data on how obesity affects acetabular cup position and spinopelvic mobility in a pre- and postoperative comparison.

Therefore, our study aimed to investigate the influence of BMI on (1) the individual segments of the spinopelvic complex (lumbar flexibility, pelvic mobility and hip motion), (2) the sagittal spinal alignment and (3) the acetabular cup position in primary THA patients in a prospective setting using standardized standing and sitting assessment pre- and postoperatively.

## Material and methods

**Fig. 1:**
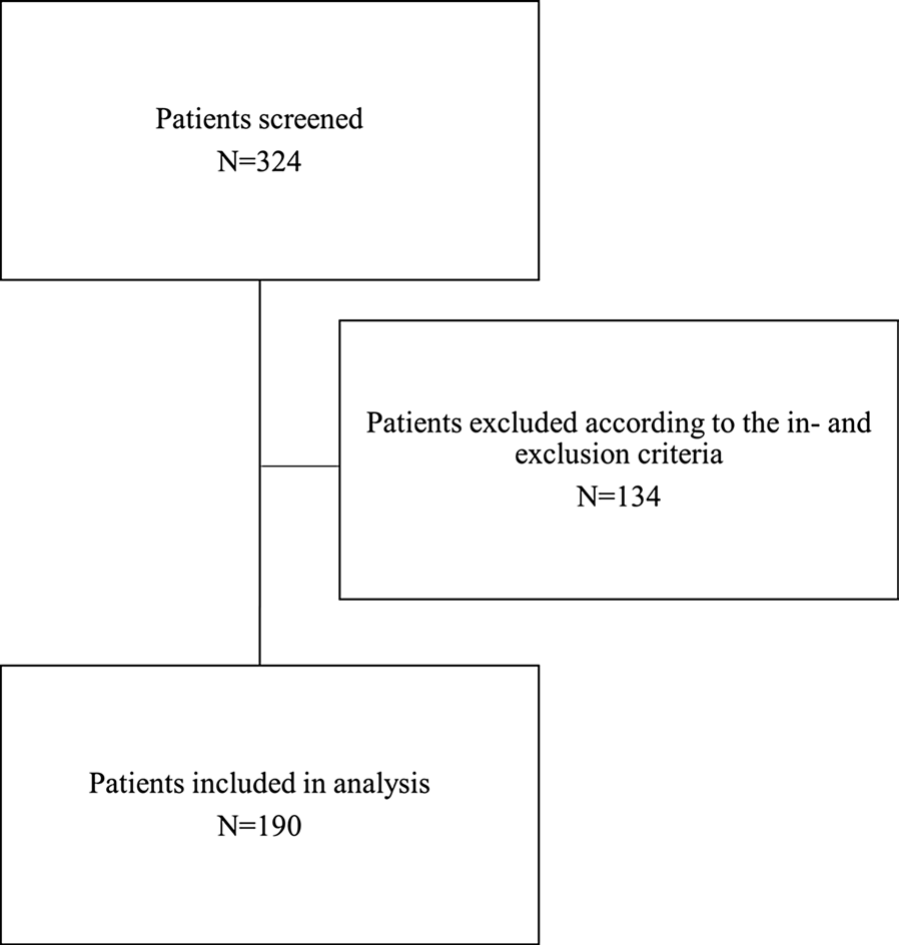
Study flow chart of screened, excluded and analyzed patients. N = 134 patients were not included in the study, because they did not match inclusion or fulfilled any of the exclusion criteria. N = 5 patients were underweighted (BMI < 18.5 kg/m2) and N = 2 patients demonstrated adipositas permagna (BMI ≥ 40 kg/m2) and were not included. 190 patients were analyzed in our investigation

A prospective radiological observational study on patients undergoing primary THA in a tertiary referral center between September 2019 and November 2020 was conducted. The investigation has been approved by the institutional ethics board (EA2/142/17) and all patients gave their written informed consent prior to study inclusion. Consecutive patients undergoing elective primary THA were included. Exclusion criteria were non-elective surgery, non-complete EOS imaging, not matching BMI definitions (underweight < 18.5 kg/m^2^ and adipositas permagna ≥ 40 kg/m^2^), bilateral THA, severe hip dysplasia with subsequent THA and femur osteotomy, any form of revision THA, ankylosing spondylitis, spinal fusion surgery at any level, osseous metastasis and pre-existing neurological conditions influencing posture. A total of 190 patients were included in the study (Fig. 1) and the data retrospectively analysed. THA was performed by four board certified surgeons in supine position via an anterolateral approach aiming for an anatomical acetabular component positioning with target values of 40° inclination and 20° anteversion with no technical assistance. Preoperative THA planning was conducted using TraumaCad (Brainlab, Munich, Germany) and the components and fixation techniques (Additional file 1: Table S1) were chosen according to the individual requirements. The indications for THA of the analyzed patients were primary osteoarthritis of the hip in 139 patients and secondary osteoarthritis in 51 patients (dysplasia of the hip: n = 19, avascular necrosis of the head: n = 14, femoroacetabular impingement cam type: n = 9, others: n = 9).

### Radiographic assessment and measurement protocols

**Fig. 2:**
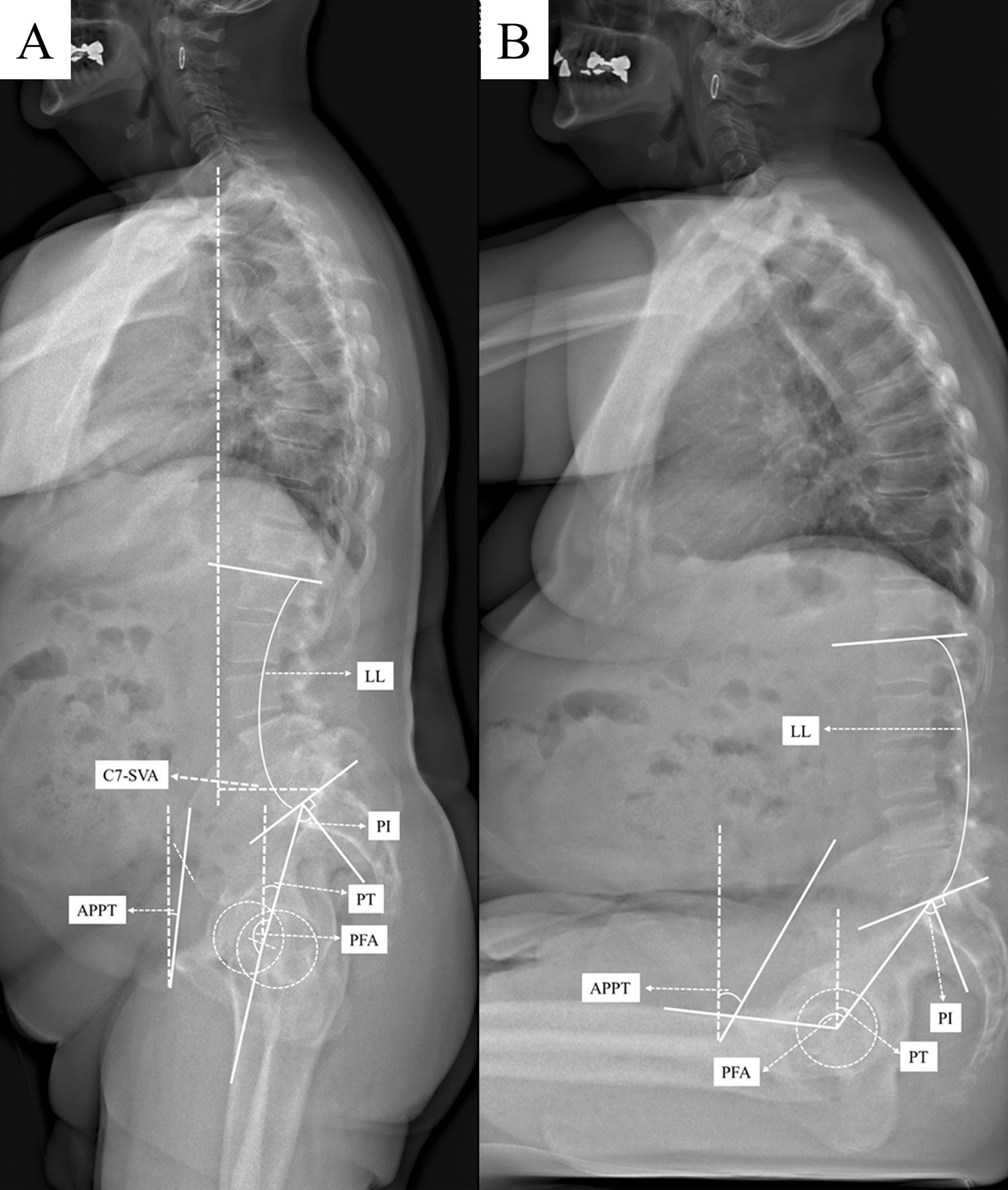
Sagittal standing (**a**) and sitting (**b**) EOS radiographs of the spine, pelvis and hip in a patient with obesity (BMI 36.3 kg/m^2^) depicting global spinal balance C7-sagittal vertical axis (C7-SVA) and spinopelvic parameter lumbar lordosis (LL),pelvic tilt (PT), pelvic incidence (PI), anterior plane pelvic tilt (APPT) and pelvic femoral angle (PFA)

A standardised measurement protocol was established in which the patients received a complete spine imaging, including the pelvis to the proximal tibia, in standing and sitting positions using biplanar low dose stereoradiography (EOS, Paris, France) within three days before and five to seven days after surgery. Patients were advised to stand naturally in the standing position, look forward and place their hands on a support with relaxed upper limbs and were instructed to sit relaxed on a height-adjustable chair without backrest, with the femur parallel to the floor. The radiographic measurements were performed by an orthopedic surgeon using Merlin Diagnostic Workcenter (Phoenix PACS, Freiburg, Germany) and a randomly selected 25% dataset was measured by a second independent orthopedic surgeon using an established randomization tool [37]. The following parameter were determined pre- and postoperatively (Fig. 2, Additional file 1: Table S2 for definition): C7-Sagittal vertical axis (C7-SVA; balance ≤ 50 mm; imbalance > 50 mm), lumbar lordosis (LL), pelvic incidence (PI), PI-LL mismatch (balance ≤ 10°; imbalance > 10°), pelvic tilt (PT), anterior plane pelvic tilt (APPT), pelvic femoral angle (PFA). Key parameters of the spinopelvic function are defined as lumbar flexibility (∆ LL = LL_standing_ − LL_sitting_), pelvic mobility (∆ PT = PT_standing_ − PT_sitting_) and hip motion (∆ PFA = PFA_standing_ − PFA_sitting_). Pelvic mobility was further defined based on ∆ PT = PT_standing_ − PT_sitting_ as stiff (∆ PT < 10°), normal (∆ PT ≥ 10°–30°), and hypermobile (∆ PT > 30°) [13]. The measurements of cup anteversion and inclination were conducted in standing and sitting anterior posterior radiographs using an established and reliable method. Inclination was defined as the angle between the line of the long axis of the ellipse and the interteardrop line and anteversion was defined by the trigonometric equation arc sine (short axis/ long axis) [38]. The patient collective was classified into three groups adapted to the WHO obesity definitions [38]: group 1: normal BMI ≥ 18.5–24.9 kg/m^2^ (n = 68), group 2: overweight ≥ 25.0–29.9 kg/m^2^ (n = 81) and group 3: obese ≥ 30–39.9 kg/m^2^ (n = 41).

### Statistical analyses

All statistical analyses were performed using SPSS Version 27 (IBM Corporation, New York, United States). Variance homogeneity was determined by Levene's test. Analysis of variance was used to determine differences between the groups in relation to BMI, variance homogeneity was given, and the post-hoc analysis according to Hochberg's GT2 (due to varying numbers of cases in each group) was applied. Spearman’s rank correlation coefficient was used to determine the interrater reliability of the radiographic measurements. A significance level of *p* < 0.05 was assumed for all tests.

## Results

Three hundred twenty-four primary total hip arthroplasty patients were screened for study eligibility and 190 patients were included and received the radiographic EOS assessment in standing and sitting position pre- and postoperatively (Fig. 1). Group 1 demonstrated a mean age of 64.7 ± 15.2 years and 45.6% female patients, group 2 revealed a mean age of 66.8 ± 11.7 years and 49.4% female patients and group 3 showed a mean age of 68.0 ± 11.3 years and 58.5% female patients with no significant differences regarding age between the groups (Group 1/2; 2/3; 1/3: *p* = 0.677; *p* = 0.953; *p* = 0.483) Interrater reliability analysis demonstrated good interobserver agreements (Additional file 1: Table S3) [39].

### BMI and acetabular cup position

Standing cup inclination demonstrated significant increases in obese group compared to normal BMI group and similar patterns in sitting assessment. Cup anteversion in standing is significantly decreased in the overweight group compared to normal BMI and a similar trend with reduced cup anteversion in sitting was observed in the overweight and obese groups (Table 1).

**Table 1:** Acetabular cup position in anteversion and inclination in standing and sitting position according to the BMI: group 1: ≥ 18.5–24.9 kg/m^2^, group 2: ≥ 25.0–29.9 kg/m^2^ and group 3: ≥ 30–39.9 kg/m^2^

BMI groups	Normal ≥ 18.5–24.9 kg/m^2^	Overweight ≥ 25.0–29.9 kg/m^2^	Obese ≥ 30–39.9 kg/m^2^	*p*-value (#1)	*p*-value (#2)	*p*-value (#3)
*Acetabular Cup Position according to BMI*						
Cup anteversion standing (°) (SD)	25.3 (7.2)	22.0 (6.7)	23.4 (6.1)	**.011**	.606	.423
Cup inclination standing (°) (SD)	40.1 (5.3)	41.4 (6.3)	43.5 (6.4)	.500	.182	**.015**
Cup anteversion sitting (°) (SD)	37.9 (7.0)	36.7 (6.6)	35.5 (6.9)	.648	.740	.217
Cup inclination sitting (°) (SD)	53.2 (9.6)	53.4 (10.3)	54.1 (10.1)	.999	.976	.953

*P*-value (#1) displayed differences between groups 1 and 2, *p*-value (#2) between groups 2 and 3 and *p*-value (#3) between groups 1 and 3. ANOVA and post-hoc analysis according to Hochberg’s GT2 were used and level of significance set at *p* < 0.05, significant values were marked in bold. SD = standard deviation

### BMI and spinopelvic function

Spinopelvic key parameter lumbar flexibility (∆ LL) was smallest pre- and postoperatively in the obese group. Pelvic mobility (∆ PT) is preoperatively the highest in the obese group, but revealed the smallest enhancement over all groups after THA leading to the smallest pelvic mobility postoperatively. ∆ LL and ∆ PT demonstrated improvements in all BMI groups after THA, while ∆ PFA decreased in all BMI groups postoperatively. The obese group showed significantly more pelvic retroversion in standing (APPT) than the normal BMI group. Pelvic retroversion in standing decreased after THA in all BMI groups with smallest changes in the obese group (PT). Standing LL was significantly smaller in the overweight and obese groups than in the normal BMI group (Tables 2 and 3).

**Table 2:** Analysis of spinopelvic complex elements lumbar flexibility (∆ LL = LL_standing_ − LL_sitting_), pelvic mobility (∆ PT = PT_standing_ − PT_sitting_) and hip motion (∆ PFA = PFA_standing_ − PFA_sitting_) and spinopelvic parameter LL, APPT, PT, PFA and PI in standing position according to the BMI: group 1: ≥ 18.5–24.9 kg/m^2^ (n = 68), group 2: ≥ 25.0–29.9 kg/m^2^ (n = 81) and group 3: ≥ 30–39.9 kg/m^2^ (n = 41) preoperatively

BMI groups	18.5–24.9 kg/m^2^ Preoperative mean (± SD)	25.0–29.9 kg/m^2^ Preoperative mean (± SD)	30–39.9 kg/m^2^ Preoperative mean (± SD)	*p*-value (#1)	*p*-value (#2)	*p*-value (#3)
*Preoperative spinopelvic parameter according to the BMI*						
∆ LL (°)	22.8 (12.0)	22.1 (12.0)	19.9 (12.8)	.977	.743	.557
∆ PT (°)	18.2 (11.2)	17.6 (10.9)	20.1 (9.3)	.982	.458	.749
∆ PFA (°)	56.4 (15.6)	57.8 (16.4)	53.9 (12.7)	.935	.484	.799
LL stand (°)	55.2 (14.0)	49.0 (12.7)	49.2 (16.5)	**.023**	**.023**	.099
PT stand (°)	14.0 (8.2)	13.4 (8.3)	15.5 (8.4)	.949	.458	.756
PFA stand (°)	179.7 (11.1)	179.1 (10.6)	179.7 (10.9)	.984	.989	1.0
APPT stand (°)	2.4 (7.6)	-0.2 (8.4)	-1.8 (8.1)	.205	.577	**.028**
PI stand (°)	56.4 (12.9)	52.5 (12.3)	56.9 (12.6)	.176	.195	.996

*P*-values indicating differences between groups 1and 2 (#1), groups 2 and 3 (#2) and groups 1 and 3 (#3). ANOVA and post-hoc analysis according to Hochberg´s GT2 were used and level of significance set at *p* < 0.05, significant values were marked in bold. SD = standard deviation

**Table 3:** Analysis of spinopelvic complex elements lumbar flexibility (∆ LL = LL_standing_ − LL_sitting_), pelvic mobility (∆ PT = PT_standing_ − PT_sitting_) and hip motion (∆ PFA = PFA_standing_ − PFA_sitting_) and spinopelvic parameter LL, APPT, PT, PFA and PI in standing position according to the BMI: group 1: ≥ 18.5–24.9 kg/m^2^ (n = 68), group 2: ≥ 25.0–29.9 kg/m^2^ (n = 81) and group 3: ≥ 30–39.9 kg/m^2^ (n = 41) postoperatively

BMI groups	≥ 18.5–24.9 kg/m^2^ Postoperative mean (± SD)	≥ 25.0–29.9 kg/m^2^ Postoperative mean (± SD)	≥ 30–39.9 kg/m^2^ Postoperative mean (± SD)	*p*-value (#1)	*p*-value (#2)	*p*-value (#3)
*Postoperative spinopelvic parameter according to the BMI*						
∆ LL (°)	25.7 (13.5)	26.0 (11.3)	25.0 (12.1)	.998	.959	.986
∆ PT (°)	22.6 (10.4)	23.0 (9.8)	21.7 (10.4)	.992	.874	.960
∆ PFA (°)	50.7 (14.0)	50.7 (12.6)	51.1 (11.4)	1.0	.998	.999
LL stand (°)	55.0 (13.4)	50.6 (12.8)	51.2 (15.9)	.153	.994	.418
PT stand (°)	10.6 (8.7)	10.6 (8.2)	13.7 (7.8)	1.0	.139	.162
PFA stand (°)	175.3 (11.1)	175.6 (8.8)	176.6 (7.8)	.998	.933	.887
APPT stand (°)	4.9 (6.7)	2.1 (7.7)	0.7 (8.6)	.089	.690	**.018**
PI stand (°)	54.7 (13.3)	51.5 (11.7)	51.1 (11.4)	.332	.098	.811

*P*-values indicating differences between groups 1and 2 (#1), groups 2 and 3 (#2) and groups 1 and 3 (#3). ANOVA and post-hoc analysis according to Hochberg´s GT2 were used and level of significance set at *p* < 0.05, significant values were marked in bold. SD = standard deviation

The preoperative proportion of stiff and hypermobile patients in terms of pelvic mobility in the obese group is lower than in the other BMI groups. There was a similar pattern in all BMI groups with a postoperative reduction in the proportion of pelvic stiffness and an increase in hypermobility after THA. The proportion of normal pelvic motility pre- and postoperatively was greatest in the obese group (Table 4).

**Table 4:** Contribution of pre- and postoperative pelvic mobility based on ∆ PT = PT_standing_-PT_sitting_ defined as stiff (∆ PT < 10°), normal (∆ PT ≥ 10°-–3°), and hypermobile (∆ PT > 30°) according to the BMI: group 1: ≥ 18.5–24.9 kg/m^2^, group 2: ≥ 25.0–29.9 kg/m^2^ and group 3: ≥ 30–39.9 kg/m^2^

Pelvic mobility (∆ PT)	Normal ≥ 18.5–24.9 kg/m^2^	Overweight ≥ 25.0–29.9 kg/m^2^	Obese ≥ 30–39.9 kg/m^2^
*Classification of pre-and postoperative pelvic mobility according to the BMI*			
Stiff (%/N)			
Pre	25.0 (17)	27.2 (22)	12.2 (5)
Post	7.4 (5)	9.9 (8)	7.5 (3)
Normal (%/N)			
Pre	60.3 (41)	56.8 (46)	78.0 (32)
Post	69.1 (47)	64.2 (52)	72.5 (29)
Hypermobile (%/N)			
Pre	14.7 (10)	16.0 (13)	9.8 (4)
Post	23.5 (16)	25.9 (21)	20 (8)

% represents the percentage contribution; N represents the absolute number of patients; pre = preoperative; post = postoperative

### BMI and sagittal spinal alignment

**Fig. 3:**
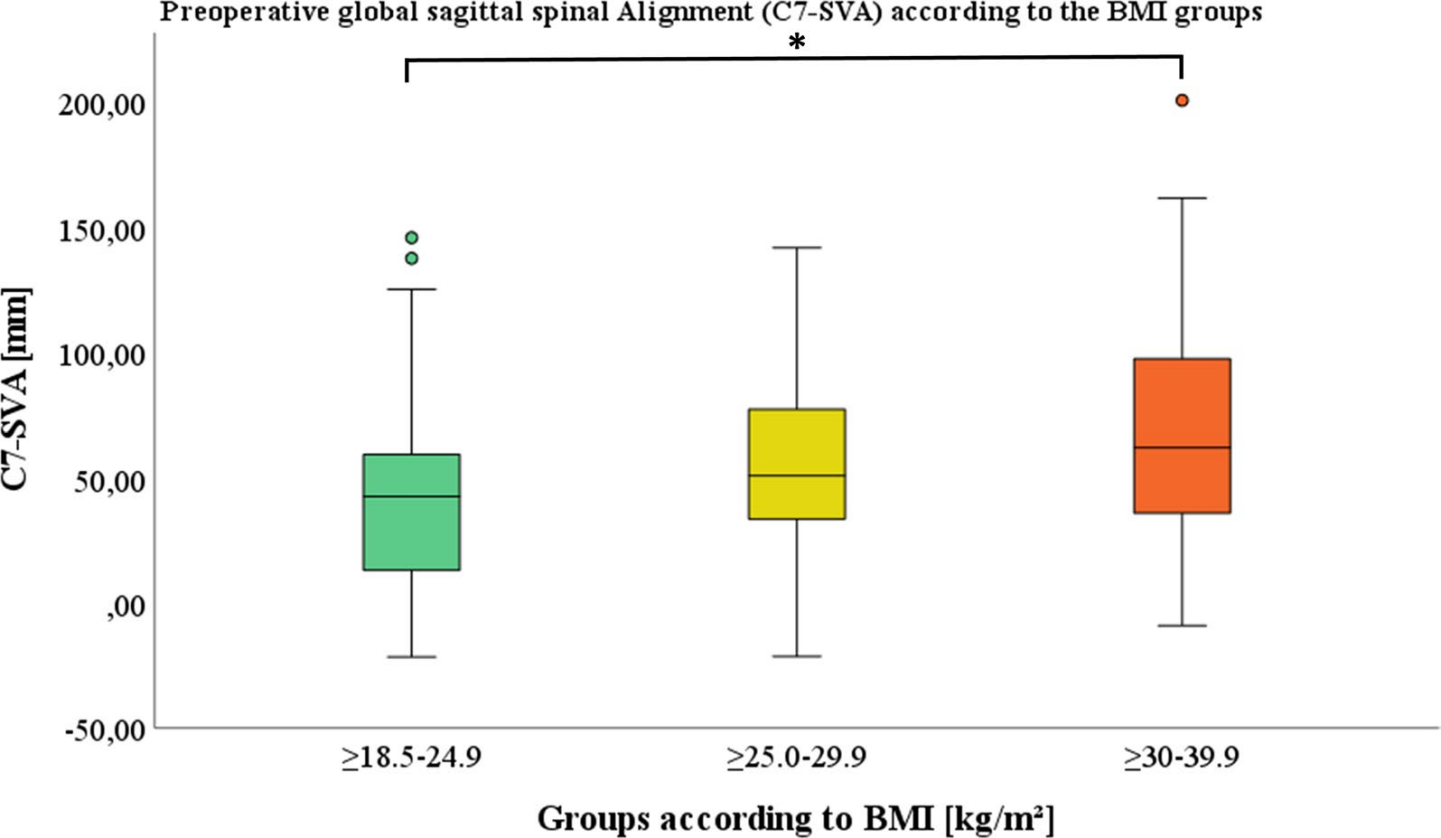
Preoperative global sagittal alignment represented by C7-SVA is depicted in relation to the defined BMI groups: group 1: ≥ 18.5–24.9 kg/m^2^, group 2: ≥ 25.0–29.9 kg/m^2^ and group 3: ≥ 30–39.9 kg/m^2^.* indicating a significant difference between group 1 and 3

**Fig. 4:**
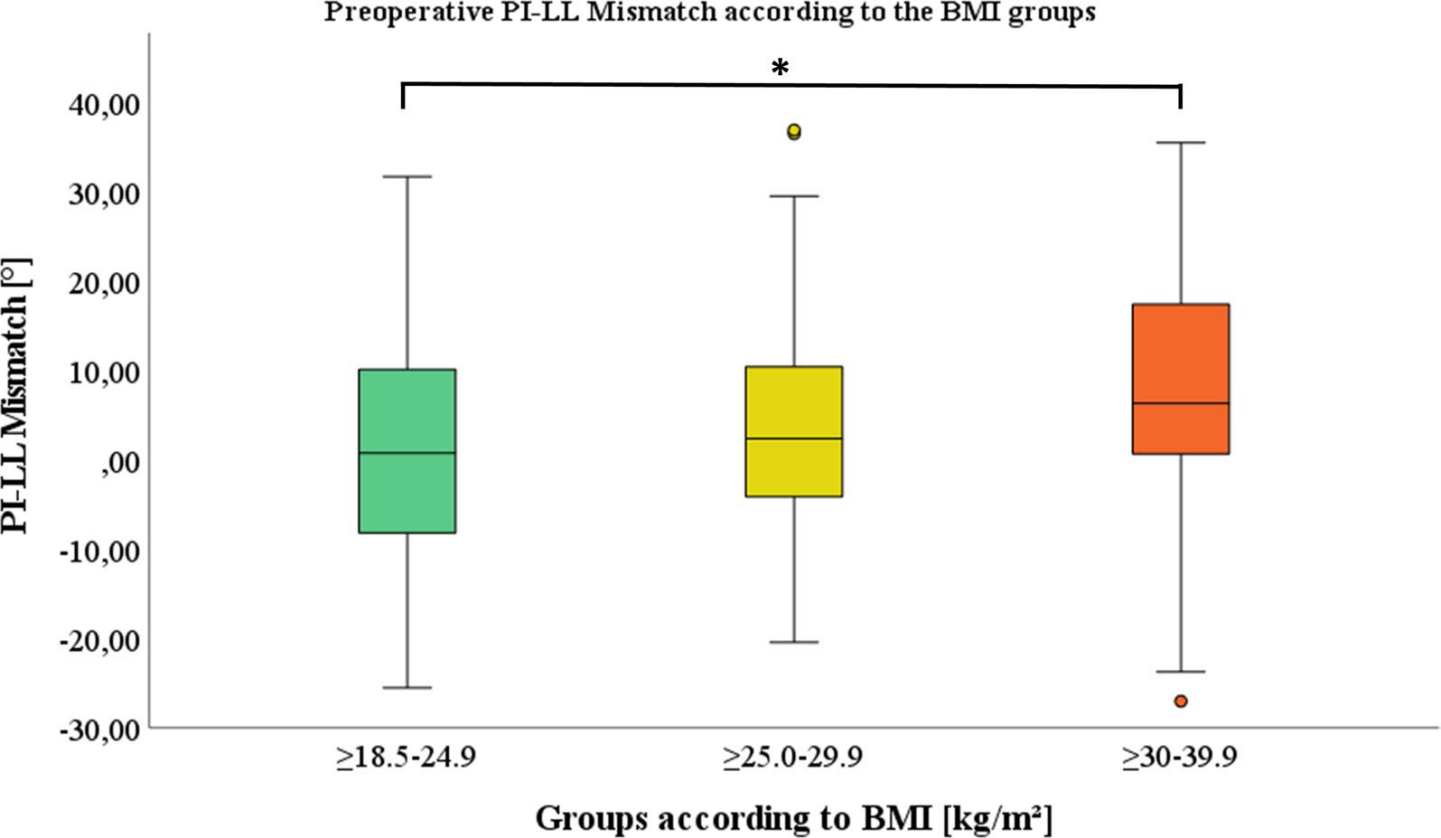
Preoperative PI-LL mismatch is depicted in relation to the defined BMI groups: group 1: ≥ 18.5–24.9 kg/m^2^, group 2: ≥ 25.0–29.9 kg/m^2^ and group 3: ≥ 30–39.9 kg/m^2^.* indicating a significant difference between group 1 and 3

Sagittal spinal alignment in both classifications (C7-SVA and PI-LL mismatch) showed significantly greater imbalance pre- and postoperatively in the obese group compared to the normal BMI group. (Figs. 3 and 4) The sagittal imbalance increased with increasing BMI. C7-SVA of > 50 mm in the overweight (Pre/Post: 54.8 mm/56.2 mm) and obese (Pre/Post 68.6 mm/65.4 mm) groups illustrated the considerable extent of imbalance (Table 5).

**Table 5:** Analysis of sagittal alignment parameter C7-central vertical axis and PI-LL mismatch preoperative (Pre) and postoperative (Post) according to the BMI: group 1: ≥ 18.5–24.9 kg/m^2^, group 2: ≥ 25.0–29.9 kg/m^2^ and group 3: ≥ 30–39.9 kg/m^2^

BMI groups	Normal ≥ 18.5–24.9 kg/m^2^ mean (± SD)	Overweight ≥ 25.0–29.9 kg/m^2^ mean (± SD)	Obese ≥ 30–39.9 kg/m^2^ mean (± SD)	*p*-value (#1)	*p*-value (#2)	*p*-value (#3)
*Spinal sagittal alignment according to the BMI*						
C7-SVA Pre (mm)	42.6 (38.1)	54.8 (36.1)	68.6 (42.7)	.155	.174	**.002**
C7-SVA post (mm)	45.4 (33.0)	56.2 (31.9)	65.4 (39.1)	.155	.403	**.010**
PI-LL mismatch pre (°)	1.2 (12.5)	3.5 (11.7)	7.7 (14.7)	.597	.251	**.032**
PI-LL mismatch post (°)	− 0.3 (12.6)	0.9 (11.6)	5.4 (13.4)	.902	.168	0.058

*P*-value (#1) displayed differences between groups 1 and 2, *p*-value (#2) between groups 2 and 3 and *p*-value (#3) between groups 1 and 3. ANOVA and post-hoc analysis according to Hochberg´s GT2 were used and level of significance set at *p* < 0.05, significant values were marked in bold. SD = standard deviation

There was a substantially higher proportion of C7-SVA imbalance in the overweight and obese groups. This pattern continued in the sagittal malalignment measurement based on PI-LL mismatch, but demonstrated an overall smaller proportion of imbalance for all BMI groups. The proportion of patients with imbalance is clearly the largest in the overweight and obese groups (Table 6).

**Table 6:** Contribution of pre- and postoperative spinal sagittal balance based on C7-SVA (balanced (≤ 50 mm) and imbalanced (> 50 mm)) and PI-LL Mismatch (balanced (≤ 10°) and imbalanced (> 10°) according to the BMI: group 1: ≥ 18.5–24.9 kg/m^2^, group 2: ≥ 25.0–29.9 kg/m^2^ and group 3: ≥ 30–39.9 kg/m^2^

BMI groups	Normal ≥ 18.5–24.9 kg/m^2^	Overweight ≥ 25.0–29.9 kg/m^2^	Obese ≥ 30–39.9 kg/m^2^
*Classification of sagittal spinal balance according to the BMI*			
C7-SVA (%/N)			
Pre			
Balance	57.4 (39)	46.9 (38)	48.8 (20)
Imbalance	42.6 (29)	53.1 (43)	51.2 (21)
Post			
Balance	55.9 (38)	48.1 (39)	41.5 (17)
Imbalance	44.1 (30)	51.9 (42)	58.5 (24)
PI-LL Mismatch (%/N)			
Pre			
Balance	72.1 (49)	74.1 (60)	56.1 (23)
Imbalance	27.9 (19)	25.9 (21)	43.9 (18)
Post			
Balance	80.9 (55)	80.2 (65)	56.1 (23)
Imbalance	19.1 (13)	19.8 (16)	43.9 (18)

% represents the percentage contribution; N represents the absolute number of patients; pre = preoperative; post = postoperative

## Discussion

The present study illustrates for the first time valuable aspects of different acetabular cup position, increased sagittal spinal malalignment and altered spinopelvic mechanics in relation to obesity based on pre- and postoperative standardized EOS assessments in 190 patients undergoing primary THA. Although some studies have already suggested an increased risk of THA dislocation after primary and revision surgery in obese patients, the causal relationship between obesity and THA dislocation has not been clarified [31–33, 40]. Investigations on the relationship of obesity and spinopelvic alignment had revealed inconsistent results [34–36].

For the first time, acetabular cup position in standing and sitting position was compared among groups stratified by BMI. A significantly larger cup inclination in standing was detected in the obese group compared to the normal BMI group. This pattern of increased inclination with increasing BMI was also evident in the sitting position. This is in accordance with the results of another study, which reported that increased BMI correlated with higher odds of inclination outside the component target zone (40° inclination and 25° anteversion) in supine pelvis radiographs in primary THA patients [41]. In this context, it should be noted that increased inclination of the acetabular components is a known risk factor for accelerated wear [42–44]. In addition, De Haan et al. reported significantly higher metal ion levels in patients with steeply inclined acetabular cups [45]. It is known, that increased acetabular cup inclination outside Lewinnek´s safe zone is a risk factor for THA dislocation [46]. Consequently, obesity is discussed as a risk factor for acetabular cup malpositioning, because the additional soft tissue permits a compromised visibility of the surgical area and a limited identification of the anatomical landmarks [47]. Two other investigations of primary THA patients showed no significant differences between obese (BMI > 30 kg/m^2^) and non-obese groups in terms of acetabular cup anteversion and inclination. Their reported values for cup anteversion and inclination in standing position were in the same range as our results (Buller/McArthur/our results: inclination obese 43.0°/40.6°/43.5°; inclination normal 41.9°/39.0°/40.1° and anteversion obese 24.9°/16.6°/23.4°; anteversion normal 23.8°/16.2°/25.3°) leading to the assumption of valid data in our investigation [41, 48]. However, cup inclination and anteversion in our investigation were found to be within the safe zone target range of inclination 30°–45° and anteversion 5°–25° in all BMI groups, and might contradict the hypothesis of BMI-associated cup malpositioning [47].

To our knowledge, this is the first study to report on the individual segments of spinopelvic function in THA patients stratified by BMI pre- and postoperatively. Overall, information on spinopelvic alignment and spinopelvic mobility in relation to BMI is scarce and so far inconclusive [35, 36, 49]. The individual segments of spinopelvic mobility (lumbar flexibility, pelvic mobility and hip motion) showed no significant differences between the BMI groups preoperatively. However, there was a trend of increased pelvic mobility and decreased hip motion in the obese group. Buckland et al. reported similar findings in their preoperative assessment of spinopelvic function in THA candidates according to the BMI, even demonstrating significant differences in pelvic mobility and hip motion from standing to sitting [49]. The increased pelvic mobility in the obese group is thought to be a compensatory mechanism for the hip motion limited by the additional soft tissue in obese patients, especially during sitting. As it is known that individual restricted segments of the spinopelvic complex are overcompensated by the other segments. This is reflected in patients with lumbar spine degeneration through increased hip motion and pelvic recruitment (“hip users”), but conversely also through limited hip motion and pelvic stiffness in patients with osteoarthritis of the hip with increased lumbar flexibility (“spine users”) [18, 50]. Accordingly Yeung et al. also reported limited hip flexion in obese THA patients in a clinical examination [51]. This preoperative observation could lead to the assumption, as postulated by Buckland, that increased pelvic mobility and consequently adequate pelvic retroversion in sitting (according to the equation every 1° of pelvic retroversion results in 0.7° functional acetabular anteversion) reduced the risk of anterior impingement and subsequent posterior THA dislocation in obese THA patients in sitting and would therefore be protective against THA instability [3, 4, 49]. Since Buckland et al. only studied THA candidates preoperatively, they were not able to make a statement about the postoperative improvement in pelvic mobility that was observed in our investigation. This improvement in pelvic mobility is most evident in the normal and overweight BMI groups, each with a postoperative increase in ∆ PT = PT_standing_ − PT_sitting_ of 4.4° and 5.4° compared to the obese group ∆ PT 1.6°. This resulted in the lowest pelvic mobility in the obese group postoperatively and relativized the assumption that obesity could be protective against THA instability, but confirmed known studies that consider obesity as a risk factor for THA instability [31–33, 49]. Nevertheless, in our study, stratified by BMI and pelvic mobility, the lowest proportion of stiff pelvic mobility was found in the obese group (12.2%) preoperatively, which supported Buckland et al. and our findings of THA candidates with increased pelvic mobility in the obese group preoperatively [49]. Furthermore the obese group revealed the largest proportion of normal classified pelvic mobility (78.0% versus 60.3% normal and 56.8% overweight BMI). Thus, we conclude, derived from our data, that obesity is not a risk factor for abnormal pelvic mobility and that the increased THA dislocation rate reported in other studies is might not due to altered spinopelvic function in obese THA patients.

So far it is unknown whether the spinopelvic alignment (in standing position) is influenced by obesity. Our study showed a significantly lower lumbar lordosis in the overweight and obese group and a significantly higher pelvic retroversion in the obese group pre- and postoperatively (APPT), whereas the other spinopelvic parameters, PT, PFA and PI showed no significant differences between the BMI groups. In the few other studies on this topic, Romero-Vargas et al. showed no significant differences between BMI stratified groups in LL, PT, SS (sacral slope) and PI in a study of 200 healthy volunteers [36]. Similarly, Buckland et al. showed no significant preoperative differences between BMI groups in THA candidates in relation to PT, PI and LL. Supporting our findings with lower LL in the overweight and obese groups, Boulay et al. reported a correlation between BMI and LL, whereas another investigation reported conflicting results with increased LL in obese patients [34, 52].

The sagittal spinal alignment is significantly influenced by obesity as we observed greater global (C7-SVA) and regional (PI-LL mismatch) sagittal spinal imbalance with increasing BMI. A distinctly larger proportion of overweight and obese patients with sagittal imbalance were detected compared to the normal BMI group. Jalai et al. also reported significant increased global imbalance (C7-SVA) in obese patients [53]. The increased sagittal malalignment in obese patients may be promoted by the additional soft tissue pulling ventrally leading to a ventral shift of the body´s center of gravity. The obese patients also exhibited a known compensatory mechanism of sagittal spinal imbalance with increased pelvic retroversion (PT and APPT) [20, 22, 54]. Accordingly another investigation reported increased PI-LL mismatch, C7-SVA and pelvic retroversion in obese patients after adult spine deformity correction surgery [55]. Increased pelvic retroversion in standing is reported as an associated factor for unfavourable pelvic mobility and acetabular component orientation [56]. Accordingly sagittal spinal malalignment in THA patients had been related to a high prevalence of excessively anteverted acetabular components [57]. The increased pelvic retroversion may lead to an enhanced risk of posterior impingement and anterior dislocation in THA patients in the standing position, as reflected in patients with ankylosing spondylitis [58, 59]. Furthermore PI-LL mismatch is reported to be associated with anterior THA impingement in the sitting position [4]. Accordingly, DelSole et al. demonstrated an increased risk of dislocation in patients with spinal sagittal deformity and concomitant THA [60]. Therefore, the reported THA instability in obese patients might be due to associated increased sagittal spinal imbalance and subsequent alterations in pelvic mechanics.

Some limitations of the study need to be addressed. EOS assessments were performed during hospitalization and only short-term follow up is presented, but long-term follow-up is planned to identify progressive changes and support us in improved understanding of THA instability linked to spinopelvic function and obesity. In our study, the relaxed seated position was selected as the functional assessment and a deep flexed seated or single leg standing position was not performed as an additional functional exercise. These functional images were not possible in the postoperative setting due to patient safety [7, 9, 50]. When evaluating the results, it needs to be considered that the implant positioning was performed in supine position, the spinopelvic assessment analyzed the relaxed seated and standing position, with known strong correlations between standing and supine position [61]. Assessing the risk of THA dislocation, both acetabular component and femoral stem positioning are relevant, in our study femoral anteversion was not examined. This leads to a potentially incomplete biomechanical representation of the instability risk and should be taken into account as a suspected bias when considering the results. The exclusion of underweight and extremely overweight patients (underweight < 18.5 kg/m^2^ and adipositas permagna ≥ 40 kg/m^2^) may have biased the results. By definition, the BMI used for the measurement of obesity cannot distinguish between the distributions of the additional soft tissue, so distortions regarding soft tissue impingement may have occurred influencing the spinopelvic mechanics. Nevertheless, BMI is the most recognized score for measuring obesity.

In conclusion, we were able to confirm that the BMI has an influence on acetabular cup positioning. Although key parameters of spinopelvic function (lumbar flexibility, pelvic mobility and hip motion) did not significantly differ depending on the BMI, there was a distinctly lower proportion of pelvic stiffness in obese THA patients. Additionally, we observed a significantly increased sagittal spinal malalignment with consequently altered pelvic mechanics (increased pelvic retroversion) in obese patients, which we suspect to be a potential cause for the increased risk of THA dislocation in these patients. These factors should be considered during preoperative planning of THA in obese patients in order to reduce the risk of dislocations, e.g. by choosing a reduced cup anteversion.

## Supplementary Information

**Additional file 1.** **Supplement Table 1.** Overview of the fixation techniques applied and the components used. **Supplement Table 2.** Measured radiological parameters with description arranged by sagittal spinal alignment and sagittal spinopelvic parameter. **Supplement Table 3.** Pre-and postoperative values and the mean of interrater reliability of the global spinal alignment and spinopelvic parameters. Spearman´s rank correlation coefficient was used.

## Data Availability

The dataset supporting the conclusions of this article is included within the article.

## Abbreviations

THA: Total hip arthroplasty
BMI: Body mass index
C7-SVA: C7-Sagittal vertical axis
LL: Lumbar lordosis
PI: Pelvic incidence
PT: Pelvic tilt
APPT: Anterior plane pelvic tilt
PFA: Pelvic femoral angle
∆ LL: Lumbar flexibility
∆ PT: Pelvic mobility
∆ PFA: Hip motion
WHO: World health organization
PI-LL mismatch: Pelvic incidence minus lumbar lordosis
