# Supplementary material for: Does obesity affect acetabular cup position, spinopelvic function and sagittal spinal alignment? A prospective investigation with standing and sitting assessment of primary hip arthroplasty patients
Source: J Orthop Surg Res. 2021 Oct 26;16:640. doi: 10.1186/s13018-021-02716-8 (PMC8547029; doi:10.1186/s13018-021-02716-8)
Supplement: Supplementary file 1 — Additional file 1. Supplement Table 1. Overview of the fixation techniques applied and the components used. Supplement Table 2. Measured radiological parameters with description arranged by sagittal spinal alignment and sagittal spinopelvic parameter. Supplement Table 3. Pre-and postoperative values and the mean of interrater reliability of the global spinal alignment and spinopelvic parameters. Spearman´s rank correlation coefficient was used. [file 13018_2021_2716_MOESM1_ESM.docx]

**Components and Fixation Techniques**

| **Component / Fixation** | **Manufacturer** |
| --- | --- |
| Cementless Fixation Technique |  |
| Allofit Cup | Zimmer Biomet, Warsaw, USA |
| TMT Cup | Zimmer Biomet, Warsaw, USA |
| R3 Cup | Smith & Nephew, London, UK |
| UHMWPE- Inlay | depends on the other components |
| Ceramic- Inlay | depends on the other components |
| Ceramic Head | Biolox Delta, CeramTec, Plochingen, Germany |
| SL-Plus MIA Standard Offset Stem | Smith & Nephew, London, UK |
| SL-Plus MIA Lateral Offset Stem | Smith & Nephew, London, UK |
| SLR-Plus Stem | Smith & Nephew, London, UK |
| Avenir Standard Offset Stem | Zimmer Biomet, Warsaw, USA |
| Avenir Lateral Offset Stem | Zimmer Biomet, Warsaw, USA |
| Avenir Complete Standard Offset Stem | Zimmer Biomet, Warsaw, USA |
| Avenir Complete High Offset/  Coxa Vara Stem | Zimmer Biomet, Warsaw, USA |

**Supplement Table 1.** Overview of the fixation techniques applied and the components used

**Radiological Parameter**

| **Radiological Parameter** | **Description** |
| --- | --- |
| C7-Sagittal vertical axis (C7-SVA) | Horizontal distance between a line from the center of the C7 vertebral body to the posterior superior corner of the sacral endplate and a plumb line from the center of the C7 vertebral body. |
| Pelvic incidence-lumbar lordosis (PI-LL mismatch) | Difference between pelvic incidence and lumbar lordosis assuming >10° difference as imbalanced. |
| Lumbar lordosis (LL) | Angle between superior endplate of L1 and superior endplate of S1. |
| Pelvic incidence (PI) | Angle between the line connecting the midpoint of the superior plate of S1 and the midpoint of the hip axis with the line perpendicular to the superior plate of S1. |
| Anterior plane pelvic tilt (APPT) | Angle between the line connecting the midpoint of both anterior superior iliac spines to the pubic symphysis, and a vertical line. |
| Pelvic tilt (PT) | Angle between the line joining the midpoint of the hip axis to the midpoint of S1superior endplate and the vertical reference line |
| Pelvic femoral angle (PFA) | Angle between the center of the hip axis to the midpoint of the superior sacral endplate and a 10 cm line from the center of the hip axis to the ventral cortex of the femur |

**Supplement Table 2**. Measured radiological parameters with description arranged by sagittal spinal alignment and sagittal spinopelvic parameter.

**Interrater reliability**

|  | *Preoperative* | *Postoperative* | *Mean (Pre-postoperative)* | |  |
| --- | --- | --- | --- | --- | --- |
| C7-Sagittal vertical axis | .614 | .571 | | .593 | |
| Lumbar lordosis | .746 | .779 | | .763 | |
| Pelvic incidence | .534 | .728 | | .631 | |
| Pelvic tilt | .900 | .816 | | .858 | |
| Anterior plane pelvic tilt | .520 | .554 | | .537 | |
| Pelvic femoral angle | .694 | .831 | | .763 | |

**Supplement Table 3.** Pre-and postoperative values and the mean of interrater reliability of the global spinal alignment and spinopelvic parameters. Spearman´s rank correlation coefficient was used.
